# Beyond Talking: We Need Effective Measures to Tackle Systemic Corruption and the Power That Allows It to Persist in Health Systems; A Response to Recent Commentaries

**DOI:** 10.15171/ijhpm.2020.21

**Published:** 2020-02-15

**Authors:** Eleanor Hutchinson, Dina Balabanova, Martin McKee

**Affiliations:** ^1^Department of Global Health and Development, London School of Hygiene and Tropical Medicine, London, UK.; ^2^Department of Health Systems and Policy, London School of Hygiene and Tropical Medicine, London, UK.

We were delighted to receive 14 responses to our editorial on corruption in health systems^1^ and thank the authors for their excellent contributions. It seems that in discussing why health systems researchers are reluctant to discuss corruption, this journal has created a window of opportunity to discuss this neglected topic. Taken together, the responses represent a condensed introduction to the field and key areas of concern.

All the authors agreed that corruption is a very serious problem for health systems and recognised the threat that it poses to countries’ progress towards universal healthcare. Many also draw attention to a considerable shift in the field including developments over the last 12 months which go some way to showing that policy-makers share the deep concerns that have been raised in this series of commentaries. Of these, the G20 Osaka Summit Declaration,^2^ the UN General Assembly Declaration on universal healthcare^3^ (both of which referred to the need to address corruption) and the ground work for establishment of a coalition of actors under ‘GNACTA’ (the Global Network on Anti-Corruption, Transparency and Accountability) led by the World Health Organization (WHO), the Global Fund, and the United Nations Development Programme^4^ seem the most important. While questions remain about whether there are enough conversations about corruption going on in the corridors of power, as pointed out by Stiernstedt,^5^ and while Lewis notes the major limitations in data on the scale and nature of the problem,^6^ these are unambiguously impressive gains in the field given the previous lack of recognition of the problem. Mackey argues that, taken together, these constitute a “policy window,” a critical space in the congested field of global public health in which action from the highest level seems possible.^7^

These changes raise new questions as researchers argue about what we can do to guide policy and practice. As Clarke writes, policy-makers must understand that corruption can become a manageable rather than intractable health system problem,^8^ serving as a warning to researchers not to get involved in “long, technical discussions over why something that should work in theory but does not work in practice.”^9^ Kohler reminds us that research should always be part of public policy-driven conversation, and we must keep our commitment to useful, applied research in mind as we find research questions, define methods, and publish and debate findings.^9^

Definitions however, will always matter when we discuss corruption – as Hussman^10^ and Gaitonde^11^ argue, demystifying them is very important. Kohler,^9^ as well as Mostert and Kaspers,^12^ each recommend the use of Transparency International’s definition, “the abuse of power for private gain.” It is a straightforward definition, gets to the point, and is well-known, making it useful for advocacy and catalysing broad-based support for actions. It also puts power right at the heart of studies of corruption. But, the problem for us is that this snappy definition does not give the weight to the systemic and the organisational drivers of corruption that a health systems approach demands. As Vian notes, we need “to focus on corruption as a health systems problem.”^13^ Huss,^14^ Fotaki,^15^ and Lu and colleagues’^16^ papers also recognise this and call for a focus on systemic or institutional corruption, which draws the system to the heart of the debate. This also speaks to what Clarke calls a public health approach to corruption,^8^ looking upstream to its determinants and incorporating early identification of risk and development of strong institutions to prevent corruption emerging. In this approach, dialogue and actions can serve to pre-empt full-blown corruption, which once entrenched is much more difficult to address.

Given these considerations, it can be helpful in some circumstances to expand Transparency International’s definition. While Gaitonde cautions against looking for a single definition of corruption, an adapted version of his 2017 definition for use with health systems seems useful: “The abuse or complicity in abuse, of public or private position, power or authority to benefit oneself, a group, an organization or others close to oneself *in a way which diverts institutions from their core aims*; where the benefits may be financial, material or non-material.” Such a definition is tangible and provides the space for unconfrontational discussion. However, as Kohler reminds, it is important to recognise that corruption perception and experience may vary in different contexts.^9^

## Anti-corruption – Broad-Based or Targeted?

Given all these considerations, how do we go about tackling corruption? Several responses asked whether it can ever be enough just to raise awareness about corruption or if researchers should focus instead on developing anti-corruption strategies? This is somewhat of a false dichotomy; anti-corruption strategies demand a nuanced understanding of corruption. But the shift in emphasis is critical, underpinning the fact that research should only ever be there to contribute towards the end goal of reducing corruption as a means to support the development of equitable, high quality health systems. So what sort of anti-corruption measures do we need? Clarke points out that the current legalistic anti-corruption measure for health systems, such as prohibition, criminalisation, legal reform and capacity building, are not up to the job,^8^ and it seems likely that this is the case in other sectors where anti-corruption interventions have also been spectacularly ineffective. Going forward, we will need well-evaluated examples of effective anti-corruption interventions. We share Clarke’s^8^ view that there is a strong case for taking a public health approach to corruption, addressing its upstream determinants. However, it is important that his advice on understanding risk and developing a prevention strategy that addresses it is not, as is often the case in practice, focussed at the micro level, with an excessive focus on individuals, which limits the scope for addressing the complex institutional and political structures that encourage corruption.

For an anti-corruption model to be successful, it has to help us to make a difference between forms of corruption that are (*a*) ‘survival corruption’ and constitute forms of problem solving (*b*) forms of corruption that appear “petty” but which have a profound impact on the health system (*c*) forms of corruption that are well-known about and part of the everyday, informal norms within a health system (*d*) more hidden forms of corruption that are underpinned by imbalances in political power. Why do we need to know these differences? Because each will require a specific approach and an array of interventions around which a political consensus can be built. Moreover, recognising that we are never likely to have enough resources to tackle all forms of corruption, especially in resource constrained settings, what we need is research that helps to identify the forms of corruption that are particularly pernicious and deleterious to the functioning of the health system and look for ways to address them. If our interest is in making sure that the health system functions effectively and maximise use and the quality of care in the system then anti-corruption needs a targeted approach.

## Power and Targeted Approaches

A new approach to anti-corruption will also have to take power and politics seriously; this element of a health systems approach to anti-corruption is often missing from the debates that often seek to present the power status-quo, and needs to be developed urgently. While strengthening governance and improving accountability and transparency, as advocated by Vian^13^ and Lewis,^6^ are fundamental requirements for effectively functioning health systems, anti-corruption measures often depend much too much on technocratic solutions which do not take politics and the influence of informal organisation, networks and norms into account. These can work in particular cases, as Lewis describes using examples from Eastern Europe^6^ but overall the evidence of effectiveness is limited.^17^ But strengthening regulatory institutions or anti-corruption bureaus is, intrinsically, a good idea but there are problems where these institutions have been captured by powerful interests. For example, in some countries, and even those considered to have well-functioning governance structures, disciplinary processes can be used to silence whistle blowers.^18^

We were surprised, therefore, that the responses included little discussion of the power and politics underlying these solutions. Reynolds was the only respondent who addressed this head-on, providing us with a way of thinking about how power operates; from violence, to coercion, sometimes limiting what can or cannot be spoken of in different fora.^19^ This is a good starting point, but we need a much better understanding of the ways in which power operates in different settings, with a detailed understanding of the historical background and political economy if we are to come up with contextually appropriate, effective anti-corruption measures.

Just thinking about power makes us question accountability models and approaches, some of these rely excessively on technology. It is often proposed that informing, empowering and supporting those at the bottom (such as community groups) will help to reduce corruption. But these groups often have the least institutionalised power and are least well-organised to take on corrupt officials. If technical interventions (new computing systems, e-governance, electronic payment systems) make it easier to see corruption, are there those with the resources, political will and power who will be able to act on this information once it is ‘captured.’ If they cannot, then we will be in the frustrating position where we know about corruption but feel unable to act on it.

We are proposing a new targeted approach to anti-corruption that takes informality seriously when considering new strategies. Drawing on political economy, our approach begins from an understanding that forms of corruption, from survival corruption to large scale political graft, emerge from the intertwining of policy, health systems (lack of) resources, and informal systems (networks, organisations and norms). It is in understanding how these intertwine that we can find innovative, targeted anti-corruption solutions. What is there in the informal systems, based on networks, alliances, and organisations, that might support anti-corruption action? Vian and others point to a potential role for civil society, although its ability to play this role varies considerably in different settings.^13^ Witvliet argues that a global anti-corruption movement is needed to set in motion collective actions that can be translated to the national level.^20^ What health systems investments would need to be made and how might policy have to be changed? Ideas of developmental governance^21^ and political settlements^22^ can help to understand these informal systems and how best to target interventions.

Yet as we ask about the actors involved, (who is involved in corruption and how, who is likely to support anti-corruption intervention, why and how?), we have noticed an extraordinary gap in the literature. There is very little in corruption and anti-corruption work in health that looks at the ways in which gender shapes corruption.^23^ Let us finish this response by calling upon researchers to look for targeted anti-corruption measures that take both the formal system and informal structures into account and think through the gendered nature of corruption in terms of how it is manifest and who it effects.

## Ethical issues

Not applicable.

## Competing interests

Authors declare that they have no competing interests.

## Authors’ contributions

EH prepared the first draft which DB and MM revised.

## Authors’ affiliations

^1^Department of Global Health and Development, London School of Hygiene and Tropical Medicine, London, UK. ^2^Department of Health Systems and Policy, London School of Hygiene and Tropical Medicine, London, UK.

## References

[1] Hutchinson E, Balabanova D, McKee M (2019). We need to talk about corruption in health systems. Int J Health Policy Manag.

[2] G20. G20 Osaka Leaders’ Declaration 2020. https://mofa.go.jp/policy/economy/g20_summit/osaka19/documents/final_g20_osaka_leaders_declaration.html. Accessed February 1, 2020.

[3] United Nations. Political Declaration of the High-level Meeting on Universal Health Coverage “Universal Health Coverage: Moving Together to Build a Healthier World.” New York: United Nations; 2019.

[4] World Health Organization (WHO). Tackling Corruption in the Health Sector to Leave no One Behind. WHO; 2019. https://www.who.int/gender-equity-rights/news/anti-corruption-transparency-accountability-in-health-systems/en/. Accessed February 1, 2020.

[5] Stiernstedt P (2019). Some things are rarely discussed in public - on the discourse of corruption in healthcare: Comment on “We need to talk about corruption in health systems”. Int J Health Policy Manag.

[6] Lewis M (2019). We need to measure and address corruption and poor governance in health systems: Comment on “We need to talk about corruption in health systems”. Int J Health Policy Manag.

[7] Mackey TK (2019). Opening the policy window to mobilize action against corruption in the health sector: Comment on “We need to talk about corruption in health systems”. Int J Health Policy Manag.

[8] Clarke D (2020). Changing the conversation, why we need to reframe corruption as a public health issue: Comment on “We need to talk about corruption in health systems”. Int J Health Policy Manag.

[9] Kohler JC (2019). I know it when i see it: the challenges of addressing corruption in health systems: Comment on “We need to talk about corruption in health systems”. Int J Health Policy Manag.

[10] Hussmann K (2019). Demystify false dilemmas to speak about corruption in health systems: different actors, different perspectives, different strategies: Comment on “We need to talk about corruption in health systems”. Int J Health Policy Manag.

[11] Gaitonde R (2019). Corruption - taking a deeper dive: Comment on “We need to talk about corruption in health systems”. Int J Health Policy Manag.

[12] Mostert S, Kaspers G (2019). all it takes for corruption in health systems to triumph, is good people who do nothing: Comment on “We need to talk about corruption in health systems”. Int J Health Policy Manag.

[13] Vian T (2019). High stakes require more than just talk: what to do about corruption in health systems: Comment on “We need to talk about corruption in health systems”. Int J Health Policy Manag.

[14] Huss R (2020). Our blind spots in the fight against health systems corruption: Comment on “We need to talk about corruption in health systems”. Int J Health Policy Manag.

[15] Fotaki M (2020). Why we must talk about institutional corruption to understand wrongdoing in the health sector: Comment on “We need to talk about corruption in health systems”. Int J Health Policy Manag.

[16] Lu HS, Ho BX, Miranda JJ (2020). Corruption in health systems: the conversation has started, now time to continue it: Comment on “We need to talk about corruption in health systems”. Int J Health Policy Manag.

[17] Gaitonde R, Oxman AD, Okebukola PO, Rada G (2016). Interventions to reduce corruption in the health sector. Cochrane Database Syst Rev.

[18] Dyer C (2015). Judge suggests steps to prevent employers unfairly referring whistleblowers to GMC. BMJ.

[19] Reynolds L (2019). Not up for discussion: applying Lukes’ power model to the study of health system corruption: Comment on “We need to talk about corruption in health systems”. Int J Health Policy Manag.

[20] Witvliet MI (2019). It will take a global movement to curb corruption in health systems: Comment on “We need to talk about corruption in health systems”. Int J Health Policy Manag.

[21] Khan MH (2013). Beyond good governance: an agenda for developmental governance.

[22] Khan MH. Political Settlements and the Governance of Growth-Enhancing Institutions. https://eprints.soas.ac.uk/id/eprint/9968. Published 2010.

[23] Hossain N, Musembi CN, Hughes J, Stern J. Corruption, Accountability and Gender: Understanding the Connections. New York: United Nations Development Programme; 2010.

